# Early recovery after surgery for radical cystectomy: comprehensive assessment and meta-analysis of existing protocols

**DOI:** 10.1007/s00345-020-03133-y

**Published:** 2020-03-02

**Authors:** F. Wessels, M. Lenhart, K. F. Kowalewski, V. Braun, T. Terboven, F. Roghmann, M. S. Michel, P. Honeck, M. C. Kriegmair

**Affiliations:** 1 Department of Urology, University Medical Centre Mannheim, Theodor-Kutzer-Ufer 1-3, 68167 Mannheim, Germany; 2 Library for the Medical Faculty, Mannheim of Heidelberg University, Theodor-Kutzer-Ufer 1-3, 68165 Mannheim, Germany; 3 Department of Anesthesiology and Critical Care Medicine, University Medical Centre Mannheim, Theodor-Kutzer-Ufer 1-3, 68165 Mannheim, Germany; 4 Department of Urology, Marien Hospital, Ruhr-University Bochum, Herne, Germany

**Keywords:** ERAS, Fast track, Cystectomy, Protocol, Systematic review, Meta-analysis, evidence-based medicine

## Abstract

**Purpose:**

Different enhanced recovery after surgery (ERAS) protocols (EP) for radical cystectomy (RC) have been published. Protocols highly differ in number of included items and specific measures.

**Materials and methods:**

A systematic review and meta-analysis on EPs in RC were performed using the databases MedLine, Cochrane Library, Web of science, and Google Scholar. The specific ERAS measures of the protocols were extracted, analyzed, and compared. Pooling of available outcome data was performed for length of stay, complications, readmission rate, and time to defecation.

**Results:**

The search yielded a total of 860 studies of which 25 studies were included in qualitative and 22 in quantitative analysis. Oral bowel preparation (BP) was omitted in 24/25 (96%) EPs, optimized fluid management was administered in 22/25 (88%) EPs and early mobilization (postoperative day 1) in 21/25 (84%). Gum chewing (*n* = 12, 46%), metoclopramide (*n* = 11, 44%), and alvimopan (*n* = 6, 24%) were the most common measures to prevent postoperative ileus. Our meta-analysis revealed a significant benefit in favor of EPs for the outcome parameters length of stay [mean difference (MD) *−* 3.46 d, 95% confidence interval (CI) − 4.94 to − 1.98, *p* < 0.01], complications [Odds ratio (OR) = 0.76, 95% CI 0.61–0.94, *p* = 0.01] and time to defecation (MD − 1.37 d, 95% CI − 2.06 to − 0.69, *p* < 0.01). Readmission rate did not show a significant difference (OR = 0.73, 95% CI 0.52–1.03, *p* = 0.07).

**Conclusion:**

Current EPs focus on omitting oral BP, early mobilization, and optimized fluid management while they differ in methods preventing postoperative ileus. Our meta-analysis revealed a benefit in introducing these protocols into clinical practice.

## Introduction

Radical cystectomy (RC) and consecutive urinary diversion as gold standard therapy for muscle invasive bladder cancer is associated with considerable morbidity and mortality [1–3]. Amongst others, postoperative ileus is one of the most common complications after RC. Furthermore, pain and delayed mobilization may induce medical complications and prolong hospital stay and rehabilitation. Therefore, enhanced recovery after surgery (ERAS) protocols (EP) have been adopted for RC to improve perioperative management.

For RC, different EPs include various measures, e.g. omitting oral bowel preparation, optimized fluid management, prevention of postoperative ileus and others [4].

Prospective studies on ERAS implementation could show that length of stay (LOS) can be reduced [5–7] although few studies found no significant difference [8]. Regarding complications after RC, the majority of prospective studies showed no significant difference for implementation of an EP [5, 9] whereas few studies could show a lower complication rate [10]. Also, time to defecation seems to be shorter after EP implementation [6, 11].

Current systematic reviews and meta-analyses focus on assessing the impact of ERAS implementation on perioperative outcome after RC [12, 13]. However, existing systematic reviews fail to address differences amongst the published protocols regarding specific ERAS measures. Consequently, the transfer of the rather general recommendations into clinical practice is impeded. Therefore, the aim of this systematic review and meta-analyses was to examine similarities and differences in specific ERAS measures between existing protocols as clinical guidance. Furthermore, we assessed the impact of ERAS implementation on LOS, complications, readmission rate, and return of bowel function after RC.

## Materials and methods

This systematic review and meta-analysis was conducted following the guidelines of Cochrane Handbook for Systematic Reviews and Interventions [14] and in line with the PRISMA statement [15] and the AMSTAR 2 criteria [16]. A review protocol was developed and registered to PROSPERO (CRD42019140214).

### Search protocol

The systematic search was performed by a librarian (V.B.) at Medical Faculty Mannheim of Heidelberg University using the following databases: MedLine through Pubmed, Cochrane Central Register of Controlled Trials in the Cochrane Library, Web of science Core Collection and Google Scholar. Google Scholar was tested using the software Publish or Perish with a reduced search strategy and the export of the first 100 hits. The other searches were finally conducted on 27th February with the following search strategy: ["Cystectomy"(Mesh) OR Cystectom*(tiab) OR "urinary diversion"(tiab)] AND ["Postoperative Care"(Mesh) OR "enhanced recovery after surgery"(tiab) OR eras protocol*(tiab) OR "enhanced recovery"(tiab) OR "enhanced protocol"(tiab) OR enhanced pathway*(tiab) OR recovery program*(tiab) OR "postoperative care"(tiab) OR "fast track"(tiab)].

Also, studies on EPs mentioned in the scanned articles were scanned and added if they were applicable.

### Inclusion criteria

Studies meeting the following criteria were included in this review:

P (population): patients with bladder cancer.

I (intervention): radical cystectomy with EP.

C (comparison): radical cystectomy without EP.

O (outcome): LOS, complication rate, time to defecation, readmission rate (at least one).

The EP had to be published in the article or had to be described in great detail. If multiple protocols were published by the same authors (e.g. after modification), the newest study / newest protocol was included. Studies, which did not provide perioperative outcomes, were still included for comparison of protocols but were excluded from meta-analysis.

All reviews were excluded but were scanned for possible relevant studies missed by our search. Articles investigating single ERAS items only were excluded as well as studies focusing on postoperative measures only. Non-English studies were also excluded.

All steps were done by two authors (F.W. and M.L.). If there were conflicts in choice of study, these were discussed and then decided together with a third party (M.K.).

### Statistical analysis

Endpoints were quantitatively summarized and pooled using review manager software (Revman version 5.3, The Cochrane Collaboration, The Nordic Cochrane Centre, Copenhagen, Denmark). For dichotomous data (e.g. complications, readmission rate) odds ratio (OR) with 95% confidence interval (CI) was calculated using the Mantel–Haenszel model. Differences for continuous data (e.g. LOS, time to defecation) were presented by mean difference with 95% CI and calculated with the inverse variance model. Data which was not reported as mean and standard deviation (e.g. in case of median and range) was transformed using the methods described by Hozo et al. [17] and Higgins and Green [18]. Random effects model was used to account for clinical heterogeneity among the studies. Heterogeneity was investigated with the *X*^2^ and *I*^2^ test and interpreted as follows: 0–40% low, 30–60% moderate, 50–90% high and 75–100% considerable [19]. Pooled analyses were visualized with Forest plots.

### Quality assessment

Quality assessment of the selected studies was performed using Newcastle Ottawa Scale (NOS) for non-randomized studies [53] and Cochrane Risk of Bias tool 2 [20] for RCTs.

### Certainty of evidence

Certainty of evidence was evaluated using the GRADE approach [21]. A summary of findings table was produced using GRADE Pro Software (McMaster University and Evidence Prime Inc, Ontario, Canada).

## Results

### Study selection

**Fig. 1:**
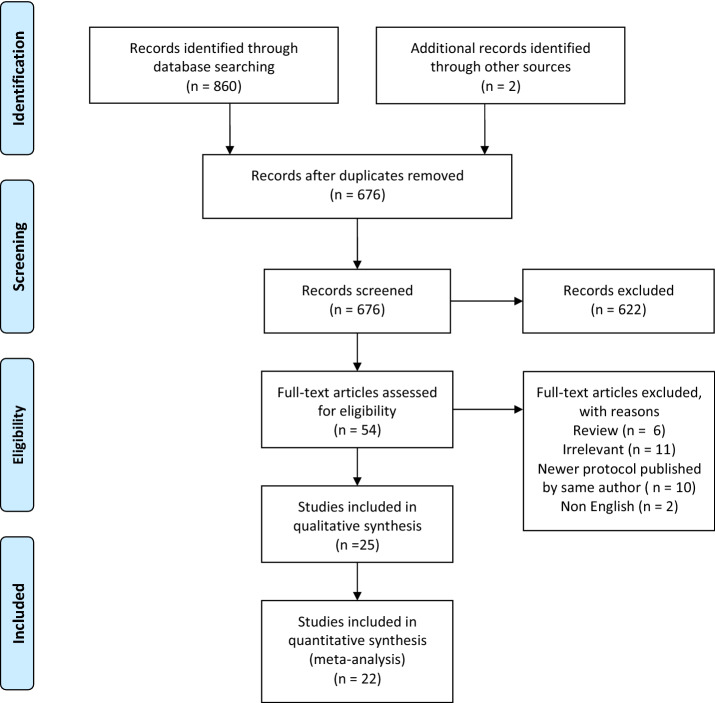
PRISMA flow diagram

The search identified 860 studies. After removal of duplications and elimination by abstract screening, 54 studies were identified for full text review. Of these studies, 25 were included in our review (see Fig. 1). These studies included 2249 patients undergoing RC with an EP.

As two studies [22, 23] had no control group (non-ERAS) and one study [24] compared two different EPs, we included 22 studies in our meta-analysis. In the analysis, 1909 patients receiving perioperative care with an EP were compared to 1917 non-ERAS patients.

### Comparison of ERAS protocols

2 randomized controlled trials (RCTs), 14 prospective and 9 retrospective studies were included (see Table 1). In 13 (52%) studies open RC was performed, in 4 (16%) studies robotic RC, in one (4%) study laparoscopic RC, in 6 (24%) studies open and robotic RC and in one (4%) study open, robotic, and laparoscopic RC.

**Table 1:** Study characteristics and preoperative counseling

Study	*n* (ERAS / Non-ERAS)	Type of study	Urinary diversion EP,* n* (%) NB, Pouch, IC, UC	Robotic surgery EP,* n* (%)	Preoperative Counseling / Optimization (spec. measures)
Altobelli et al. [14]	207/177	Retrospective	71/6/130/0 (34/3/63/0)	31 (15)	Yes (not specified)
Baack Kukreja et al. [1]	79/121	Prospective (ERAS only) single center non-randomized	7/1/113/0 (6/4/90/ 0)	44 (56)	Yes (stoma, tox cessation, comorbidities, nutrition, physical training)
Brockmann et al. [25]	152/147	Prospective (ERAS only)	n.s./n.s./120/n.s (n.s./n.s./79/n.s.)	56 (37)	No
Casans-Francés et al. [2]	41/97	Retrospective	11/0/30/0 (27/0/73/ 0)	0 (0)	Yes (stoma, tox cessation, comorbidities, nutrition)
Cerruto et al. [26]	9/13	Prospective (ERAS only) single center non-randomized	9/0/0/0 (100/0/0/0)	0 (0)	Yes (comorbidities, nutrition)
Collins et al. [5]	135/86	Prospective (all) single center non-randomized	38/0/97/0 (28/0/72/0)	135 (100)	Yes (stoma, tox cessation, comorbidities, physical activity, social)
Djaladat et al. [9], Djaladat et al. [9, 27]	110/484	Prospective (ERAS only) single center non-randomized	105/11/53/0 (62/7/31/0)	0 (0)	Yes (tox cessation, social)
Dutton et al. [22]	165	Retrospective	34/0/131/0 21/0/79/0	0 (0)	Yes (stoma, comorbidities, nutrition, physical activity, social)
Frees et al. [3]	10/13	Prospective single center randomized controlled	3/0/7/0 (30/0/70/0)	0 (0)	Yes (not specified)
Jensen et al. [17]	107	Prospective single center (RCT for factor mobilization)	12/3/92/0 (12/2/86/0)	25 (23)	Yes (stoma, comorbidities, nutrition, physical activity)
Koupparis et al. [15]^a^	102 robotic (For MA: open 52/52)^a^	Prospective single center non-randomized (MA retrospective)	11/0/91/0 (11/0/89/0) MA: 4/0/48/0 (7/0/93/0)	102 (100) MA:0 (0)^a^	Yes (stoma, social)
Lin et al. [7]	144/145	Prospective multicenter randomized controlled	53/0/91/0 (37/0/63/0)	7 (5); Laparascopic: 112 (78)	No
Liu et al. [28]	84 / 176	Retrospective	0/0/84/0 (0/0/100/0)	0 (0)	No
Maffezzini et al. [29, 30]	71/40	Prospective (ERAS only) single center non-randomized	23/27/31/0 (32/38/30/0)	0 (0)	Yes (comorbidities, nutrition)
Mukhtar et al. [11]	51/26	Prospective (all) single center non-randomized	3/0/48/0 (6/0/94/0)	0 (0)	Yes (nutrition)
Palumbo et al. [8]	74/40	Prospective (all) single center non- randomized	22/0/24/25 (30/0/33/34)	0 (0)	No
Pang et al. [31]	393/60	Prospective (all) single center non-randomized	25/0/368/0 (6/0/94/ 0)	28 (7)	Yes (stoma, tox cessation, comorbidities, physical activity)
Patel et al. [24]	116/143	Retrospective, comparison surgical vs. multidisc. ERAS	19/5/92/0 16/4/79/ 0	0 (0)	No
Persson et al. [32]	31/39	Prospective (all) single center non-randomized	5/0/26/0 (17/0/83/0)	0 (0)	Yes (not specified)
Rivas et al. [20]	19/28	Retrospective	4/0/15/0 (21/0/79/0)	Laparascopic: 19 (100)	Yes (not specified)
Saar et al. [8]	31/31	Prospective (all) single center non-randomized	8/0/23/0 (26/0/74/0)	31 (100)	No
Semerjian et al. [33]	56/54	Prospective (ERAS only) single center non-randomized	3/0/53/0 (5/0/95/0)	8 (14)	Yes (not specified)
Smith et al. [34]	27/69	Retrospective	0/0/27/0 (0/0/100/0)	0 (0)	Yes (stoma, nutrition)
Tan et al. [10]	40/210	Prospective (all) single center non-randomized	11/0/39/0 (22/0/78/0)	40 (100)	Yes (stoma, comorbidities)
Wei et al. [35]	91/101	Retrospective	3/0/82/6 (3/0/90/7)	0 (0)	Yes (tox cessation, nutrition)

*MA* meta−analysis

^a^Non ERAS−patients underwent only open cystectomies therefore MA was performed open non ERAS vs open ERAS not including robotic patients for better comparability

### Preoperative counseling

Preoperative counseling was included in 21 protocols. The extent was variable, but items included were e.g. consultation of a stoma therapist (*n* = 9), optimizing medication/comorbidities (*n* = 8), cessation of alcohol/smoking (*n* = 6), and others (see Table 1).

### Bowel function

24/25 protocols omitted oral bowel preparation (96%) whereas retrograde bowel preparation was still performed in 7 EPs (28%). Carbohydrate loading was administered in 18/25 EPs (72%) and 12 (48%) included postoperative gum chewing. In 23/25 (92%) protocols nasogastric tube was removed at the end of operation. 21/25 (84%) protocols included medical bowel stimulation: here, metoclopramide was the most common drug used in 11 (44%) protocols, whereas alvimopan and neostigmine were only employed in 6 (24%) and 2 (8%) protocols, respectively. Further oral and rectal medication included magnesium (*n* = 5, 20%) and rectal enema (*n* = 5, 20%, for details see Table 2, 3).

**Table 2:** Bowel management

Study	Preoperative bowel preparation (BP)			Prevention of ileus								
				Oral					i.v			Rectal
	Oral BP	Rectal BP	Carbohydrate loading	No NGT postop	Gum chewing	Mg_2+_	Alvimopan	Others	MCP	Neostigmin	Others	enema
Altobelli et al. [36]	−	−	−	−	−	−	+	−	−	−	−	−
Baack Kukreja et al. [1]	−	−	+	+	+	−	+	+	+	( +)	−	( +)
Brockman et al. [25]	−	−	−	+	−	−	+	−	−	−	−	−
Casans-Francés et al. [2]	−	−	+	+	+	−	−	+ (Lactulose)	+	−	−	−
Cerruto et al. [26]	−	+	+	+	+	−	−	−	+	−	−	( +)
Collins et al. [5]	−	−	+	+	+	−	−	−	+	−	−	−
Djaladat et al. [9], Djaladat et al. [9, 27]	−	−	+	+	−	+	+	+ (Lactulose)	( +)	+	−	−
Dutton et al. [22]	−	−	+	+	−	−	−	−	+	−	−	−
Frees et al. [3]]	−	−	+	+	+	−	−	−	+	−	−	−
Jensen et al. [23]	−	+	+	n.s	−	−	−	n.s	−	−	n.s	−
Koupparis et al. [37]-	−	−	+	+	+	−	−	−	+	−	−	−
Lin et al. [7]	+ (Laxative)	−	−	+	−	−	−	−	−	−	+	( +)
Liu et al. [28]	−	−	+	+	+	+	−	−	−	−	−	−
Maffezzini et al. [29, 30]	−	+	−	−	−	−	−	−	−	−	( +)	−
Mukhtar et al. [11]	−	−	+	+	−	−	−	−	−	−	−	−
Palumbo et al. [6]	−	+	+	+	−	−	−	−	−	−	−	−
Pang et al. [31]	−	−	+	+	+	−	−	−	−	−	−	( +)
Patel et al. [24]	−	−	−	+		−	+	−	−	−	−	−
Persson et al. [32]	−	−	+	+	−	−	−	+ (Laxative)	−	−	−	−
Rivas et al. [38]	−	+	−	+	+	−	−	−	+	−	−	−
Saar et al. [8]	−	+	+	+	−	+	−	−	+	−	−	+
Semerjian et al. [33]	−	−	+	+	−	−	+	−	−	−	−	−
Smith et al. [34]	−	−	+	+	+	+	−	−	−	−	+	−
Tan et al. [10]	−	−	+	+	+	+	−	−	+	−	−	−
Wei et al. [35]	−	−	−	+	+	−	−	−	−	−	−	−

*+* regularly used, *(+)* used depending on clinical outcome, − not used, *n.s.* not stated, *MCP* metoclopramide

**Table 3:** Anesthesia and pain management

Study	No		Anesthesia			Pain management		
		Antibiotic duration	Avoid long acting sedative	Optimized fluid management	Prevention of PONV	Epidural	Regional Infiltration	Non-epidural pain medication
Altobelli et al. [36]	207	24 h	+	+	n.s	+	-	n.s
Baack Kukreja et al. [1]	79	24 h	n.s	+	n.s	+ (open)	+ (lap)	NSAI + paracetamol ± weak opioids ± narcotics
Brockman et al. [25]	152	SD	n.s	+	n.s	+	−	n.s
Casans-Francés et al. [2]	41	SD	+	+ (Protocol by Feldheiser et al. [39])	+ (Apfel scale)	+	–	NSAI
Cerruto et al. [26]	9	SD	+	+	+	+	+	NSAI
Collins et al. [5]	135	SD	+ (spinal)	+	n.s	−	−	NSAI + opioid
Djaladat et al. [9], Djaladat et al. [9, 27]	196	stent removal	+	+	n.s	−	−	NSAI + Paracetamol
Dutton et al. [22]	165	SD	n.s	+	n.s	−	+ (RSC)	NSAI + weak opioid
Frees et al. [3]	12	n.s	n.s	+ (Doppler monitoring)	n.s	+	−	NSAI ± opioid
Jensen et al. [23]	57	SD	n.s	n.s	n.s	−	−	NSAI ± opioid
Koupparis et al. [37]-	102	n.s	n.s	+	+	+	−	Paracetamol + opioid
Lin et al. [7]	144	SD	n.s	n.s	n.s	−	−	n.s
Liu et al. [28]	84	SD	+	+	+	+	−	n.s
Maffezzini et al. [29]	71	drain removal	n.s	+	n.s	+	−	n.s
Mukhtar et al. [11]	51	n.s	+	+	+	+	−	non-opioid
Palumbo et al. [6]	74	POD 4	+	+ (Ringer’s acetate solution 1–2 mg/kg/h)	n.s	−	−	Paracetamol + diclofenac ± weak opioid
Pang et al. [31]	393	24 h (m) 48 h (f)	+	+ (< 1L before bladder removal, use of vasopressors)	+	−	+ (RSC)	Paracetamol
Patel et al. [24]	116	n.s	n.s	+ (minimally invasive volume monitor)	n.s	+	−	Paracetamol
Persson et al. [32]	31	SD	+	+	+	+	−	n.s
Rivas et al. [38]	19	SD	+	+	n.s	+	−	Avoid opioid
Saar et al. [8]	31	SD	-	-	−	−	−	diclofenac + NSAI ± opioids
Semerjian et al. [33]	56	24 h	+	+ (125-200 ml Ringer/h ± bolus depending on losses, phenylephrine)	+	+ (open)	+ (Robotic: TAP-Block)	Paracetamol + gabapentin + tranSDermal lidocaine ± weak opioid ± opioid
Smith et al. [34]	27	n.s	n.s	+ (esophageal doppler)	n.s	−	+ (RSC)	n.s
Tan et al. [10]	40	n.s	+ (spinal)	+ (esophageal doppler)	n.s	−	−	NSAI + Paracetamol ± opioid
Wei et al. [35]	91	n.s	n.s	+ (vascular pressure)	n.s	+	+ (RSC)	intravenous opioid on demand

*+* used, *−* not used, *n.s.* not specified, *SD* single dose, *RSC* rectus sheath catheter, *TAP* transversus abdominis plane block

Postoperative early oral feeding (EOF) was part of 24/25 (96%) EPs. However, early was defined differently: 12 (48%) studies started EOF latest on second postoperative day, whereas EOF was started after second postoperative day in the other EPs (*n* = 13, 52%).

### Mobilization

Early mobilization (POD 1) was recommended in 21/25 (84%) EPs, 8 (32%) protocols proposed a mobilization on day of operation.

### Anesthesia and pain management

Furthermore, we compared the EPs regarding anesthesia and pain management as shown in Table 2. Avoidance of long acting sedatives was stated in 13/25 (52%) protocols. An optimized fluid management was used in 22/25 (88%) protocols, but with different measures and types of monitoring to achieve this (see Table 2 for details), e.g. 4 (12%) EPs used Doppler guided fluid management.

For pain management, epidural anesthesia was regularly used in 15 (60%) protocols, in 6 (24%) protocols local infiltration was applied. Detailed description of additional oral pain medication was given in 18 (72%) protocols and consisted mainly of nonsteroidal anti-inflammatory drugs (NSAI) with or without opioid.

### Meta-analysis on the effect of ERAS on postoperative outcome

**Fig. 2:**
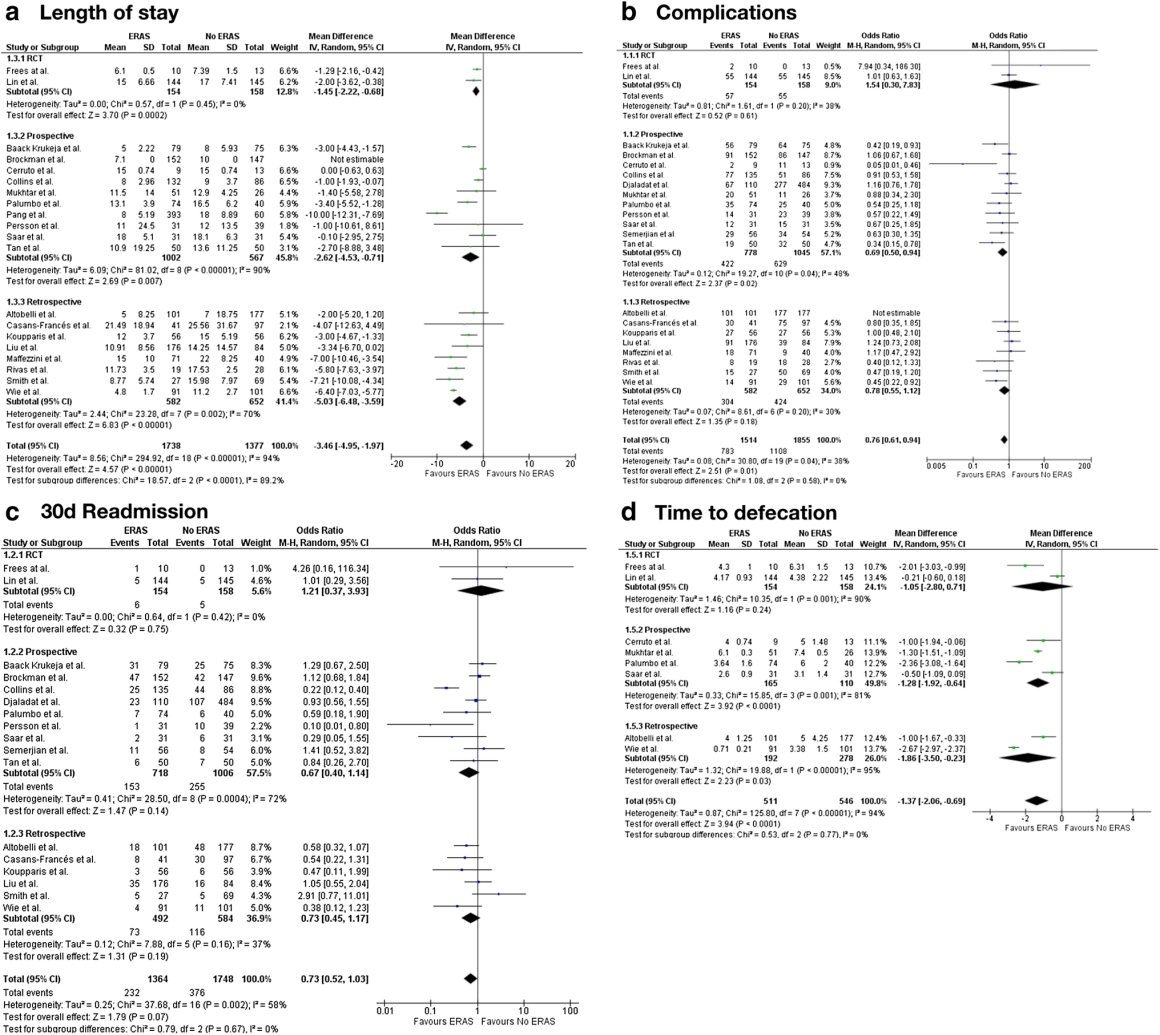
Meta-analysis of perioperative outcome

We performed a meta-analysis on the outcome parameters LOS, time to defecation, complication rate, and readmission rate including 22 studies (see Fig. 2).

20 studies reported on LOS (see Fig. 2a). Pooled data showed a shorter LOS in favor of ERAS with an estimated mean difference of 3.46 days (95% CI − 4.94 to − 1.98, *p* < 0.01). This trend was significant in all subgroups (retrospective, non-randomized prospective studies, RCTs). Heterogeneity was high (*I*^2^ = 94%).

Readmission rate (see Fig. 2b), which was reported by 17 studies, showed no significant difference in the pooled data of all studies (OR = 0.73; 95% CI 0.52–1.03, p = 0.07) or in any of the subgroups. Heterogeneity was moderate (*I*^2^ = 57%).

Regarding complications (see Fig. 2c), 21 studies reported on this outcome parameter. Odds ratio (OR) of the pooled data was 0.76 (95% CI 0.61–0.94, *p* = 0.01) in favor of ERAS. This was statistically significant in non-randomized prospective studies (*p* = 0.02) but not in the pooled RCTs (*p* = 0.62) and retrospective studies (*p* = 0.61). Heterogeneity was moderate (*I*^2^ = 38%).

Time to defecation was reported by eight studies. Here, time to defecation was shorter if an ERAS protocol was in practice (see Fig. 2d). Most studies showed a trend towards a shorter time to defecation, but the extend and their significance level was heterogenous. Pooled data from all studies showed an earlier defecation with an estimated mean difference of 1.37 days (95% CI − 2.06 to − 0.69, *p* < 0.01). This trend was significant in non-randomized studies (*p* = 0.03 for retrospective and *p* < 0.01 for prospective studies) only. Heterogeneity was high (*I*^2^ = 94%).

### Quality assessment

We performed a quality assessment of the investigated studies (see Appendix, Table 4). Seven of the non-randomized studies got 0/2 stars for comparability, six studies got 1/2 stars as the described criteria were not met. Regarding the two included RCTs, both showed some concerns to be biased, mainly because in both studies no blinding in data acquisition was practiced.

In Table 5 (Appendix), findings are summarized, and certainty of evidence is shown, which is low for LOS and very low for complication rate, time to defecation, and readmission rate.

## Discussion

Standardized EPs are increasingly implemented in urological surgery and especially for radical cystectomy [4]. Studies could prove their merit in improving perioperative outcome [12].

Accordingly, our meta-analyses could show a significant difference between standard of care and ERAS protocols for LOS, time to defecation and complications in favor of EP. Remarkably, readmission rate showed no significant difference in the pooled data.

LOS was significantly shorter in favor of ERAS in the pooling of all patients as well as in all subgroups (RCTs, prospective, retrospective studies). This is concordant to colorectal cancer studies and seems to be a proven benefit of the introduction of EPs.

Analyses including all studies could show a lower overall complication rate. However, there was no significant difference in any of the RCTs or in the group of retrospective studies, therefore the assumption that ERAS can reduce complications has to be considered with caution.

One of the most common complication after RC is postoperative paralytic ileus [40]. In our meta-analysis, a shorter time to defecation in comparison to traditional regimes in pooling of all patients could be shown. Still, in the RCT-group only Frees et al. [3] could show a significantly shorter time to defecation, whereas the RCT with the larger cohort by Lin et al. [7] as well as pooling of the two RCTs showed no significant difference.

In addition, our meta-analysis showed no significant difference in readmission after protocol implementation.

Above mentioned findings are partly concordant to other meta-analyses published on this topic [12, 41]. We were able to support their findings on LOS and in the pooling of all patients for complications and time to defecation with an even larger selection of studies in comparison to the mentioned reviews. In our opinion, additional RCTs are needed to further define the role of EPs regarding complications, time to defecation and occurrence of ileus due to the unclear results of the two included RCTs. In conclusion, EPs can reduce LOS and might reduce complication rate and time to defecation and can therefore be recommended. However, certainty of evidence of the present findings has to be considered very low to low (see Table 5) due to limited cohort size of existing RCTs (only one RCT with > 100 patients) and high risk of bias for the included non-randomized studies.

Given the high variability between published protocols, it remains challenging for clinicians to identify appropriate measures for clinical implementation. We could show that protocols differ in number and manner of included ERAS items. Furthermore, the implementation of every EP item is usually impossible in every patient rendering the assessment of EPs and especially the value of each single item difficult [31]. There are recommendations by the ERAS-Society regarding the content of a protocol for RC [4]. Although these recommendations are mostly evidence-based, some of the items were not investigated in patients undergoing RC but CRC [4]. Moreover, level of evidence is low for some of these recommendations. In addition, some of them are given in a rather general way and therefore can be implemented in different ways.

For example, the recommendation on preventing postoperative ileus is fairly general comprising only two specific measures: Gum chewing and oral magnesium [4]. Especially, for gum chewing the level of evidence is high with two RCTs showing that this measure can improve postoperative bowel function in patients undergoing radical cystectomy [42]. Regardless of this, only 48% of the investigated EPs in our analysis included this item. Beyond that, other pharmaceutic measures such as intravenous stimulation with metoclopramide (44%), or alvimopan (24%), neostigmine (8%) or others are being used to improve bowel function, even though of those mentioned only alvimopan has been proven effective in published studies [43]. The importance of improving gut motility is also shown by the fact that 52% of the protocols included more than one pharmaceutical (incl. gum chewing) method to prevent postoperative ileus. Remarkably, almost every protocol has a different approach to this item. This underlines the need for clear recommendations and further evidence. Summarizing our systematic review, gum chewing, metoclopramide, alvimopan, and magnesium are the most frequently applied measures to prevent postoperative ileus. In regard to the recommendation of the ERAS society gum chewing, alvimopan and magnesium can be suggested for clinical implementation, whereas no distinct consensus exists for i.v. medication.

Carbohydrate loading is well proven by RCTs in CRC [44]. It can shorten LOS and improve gut function [45] by decreasing insulin resistance and thirst (and is also safe in diabetic patients). Since 72% of identified protocols used this item and its clinical implementation seems to be simple, it can be endorsed for further EPs.

High accordance in omitting preoperative oral bowel preparation could be shown in our systematic review (24/25 EPs, 96%). RCTs on omitting bowel preparation in RC [46] and CRC [47] demonstrated the safety of this measure and it can therefore be recommend.

Postoperative immobilization has several negative effects as it can lead to pulmonary and thromboembolic complications [48]. In the selected EPs, 86% postulated early mobilization on POD 0 or 1. Although not specifically studied for RC, it is advisable to include this item.

Perioperative prophylactic antibiotics mostly consisted of 24-h (*n* = 11, 44%) or single dose administration (*n* = 4, 16%). At the moment no clear recommendation on duration and exact type of antibiotic is given which leads to heterogenous regimes duration of prophylactic antibiotic therapy [49, 50], therefore just a general recommendation to use prophylactic antibiotics can be given.

The positive effect of a multidisciplinary concept in comparison to an exclusively surgical concept was shown by Patel et al. [24]. They were able to show that especially by adding optimized fluid management and epidural anesthesia, transfusion rate and nausea could be reduced. In our review, in 88% of the protocols intraoperative optimized fluid management was implemented. As Pillai et al. showed a benefit for postoperative gastrointestinal function in their RCT on optimized fluid management in RC [51], this can be considered as one of the key elements in the multimodal ERAS approach. Yet, implementation is heterogenous as different strategies, especially different ways to monitor volume status, were described in the protocols (e.g. Doppler guided, vascular pressure, fixed protocols). Recent studies preferred Doppler guided monitoring although comparative studies are missing [51]. Of the included studies 4/25 (16%) used esophageal Doppler to monitor volume status. An example of a detailed protocol on Doppler guided optimized fluid management is given by Feldheiser et al. [39].

Limitations of our study result from the rather low level of evidence of the existing body of literature on EPs for RC. There were only two RCTs to be included, with one study assessing 25 patients only [3]. To provide a comprehensive overview over current evidence, non-RCTs were also regarded. Consequently, the quality of data on which the meta-analysis is based was low. Therefore, the results of our analysis should be interpreted with caution (Table 5). Further high quality RCTs are needed to confirm our findings. Also, heterogeneity (*I*^2^) in our meta-analysis has to be mentioned, which was high. Due to this fact we used a random effects model. Moreover, besides assessing the effect of ERAS implementation on postoperative outcome, we performed a comprehensive comparison of the different ERAS items. This can support clinicals in identifying suitable measures for clinical implantation and definition of their individual ERAS protocols.

## Conclusion

EPs can reduce LOS, complications and can shorten time to defecation without an increased readmission rate. Current protocols include a high number of multidisciplinary measures to achieve this improved outcome, which are summarized in this study.

## Figures and Tables

**Fig. 3:**
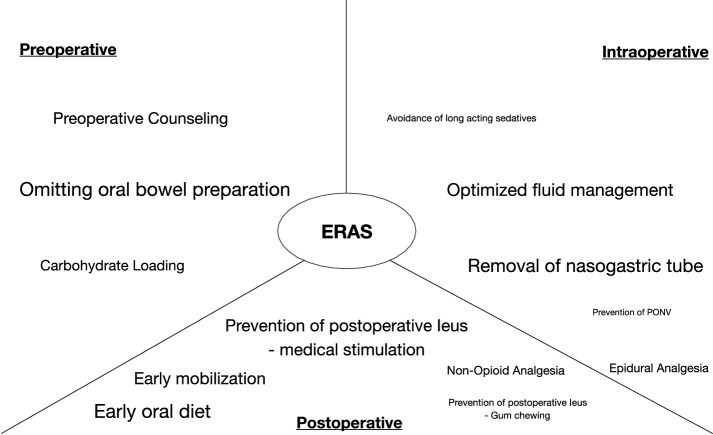
Included ERAS items (size correlates to frequency of use in EP)

## Appendix

See Fig. 3, Tables 4, 5.

**Table 4:** Quality assessment of included studies

Non-randomized Study	Type	Selection				Comparability	Outcome		
	Retrospective / Prospective / RCT	Representativeness of the exposed cohort	Selection of the non-exposed cohort	Ascertainment of exposure	Demonstration that outcome of interest was not present at start of study	Comparability of cohorts on the basis of the design or analysis	Assessment of outcome	Was follow-up long enough for outcomes to occur	Adequacy of follow up of cohorts
Altobelli et al. [36]	retrospective	*****	*****	*****	*****	**–**	*****	*****	*****
Arumainayagam et al. [52], Koupparis et al. [37]	retrospective	*****	*****	*****	*****	******	*****	*****	*****
Baack Kukreja et al. [1]	ERAS prospective	*****	*****	*****	*****	******	*****	*****	*****
Brockman et al. [25]	ERAS prospective	*****	*****	*****	*****	**–**	*****	**–**	*****
Casans-Francés et al. [2]	retrospective	*****	*****	*****	*****	***-**	*****	**–**	*****
Cerruto et al. 2014 [26]	ERAS prospective	*****	*****	*****	*****	******	*****	*****	*****
Collins et al. [5]	Prospective	*****	*****	*****	*****	**–**	*****	**–**	*****
Djaladat et al. [27]	ERAS prospective	*****	**-**	*****	*****	**–**	*****	*****	*****
Liu et al. [28]	retrospective	*****	*****	*****	*****	***-**	*****	**–**	*****
Maffezzini et al. [20]	ERAS prospective	*****	*****	*****	*****	**–**	*****	**-**	*****
Mukhtar et al. [11]	Prospective	*****	*****	*****	*****	******	*****	**–**	*****
Palumbo et al. [6]	Prospective	*****	*****	*****	*****	******	*****	*****	*****
Pang et al. [31]	Prospective	*****	*****	*****	*****	**–**	*****	*****	*****
Persson et al. [32]	Prospective	*****	*****	*****	*****	******	*****	**–**	*****
Rivas et al. [38]	Retrospective	*****	*****	*****	*****	**-***	*****	**–**	*****
Saar et al. [8]	Prospective	*****	*****	*****	*****	******	*****	**–**	*****
Semerjian et al. [33]	ERAS Prospective	*****	*****	*****	*****	***-**	*****	**–**	*****
Smith et al. [34]	Retrospecitve	*****	*****	*****	*****	******	*****	*****	*****
Tan et al. [10]	Prospective	*****	**-**	*****	*****	**–**	*****	*****	*****
Wei et al. 2018 [35]	Retrospective	*****	*****	*****	*****	**-***	*****	*****	*****

RCT	Type	RoB Randomization	RoB Deviations from intervention	RoB Missing otcome	RoB Measurement outcome	RoB Selection of reported result		Risk of Bias	
Frees et al. [3]	RCT	Some concerns	Low	Some concerns	Some concerns	Some concern		Some concerns	
Lin et al. [7]	RCT	Some concerns	Low	Low	Some concerns	Some concerns		Some concerns	

*RoB *risk of bias

This table shows a quality assessment for non-randomized and randomized studies. For non-randomized studies Newcastle Ottawa scale was used. Here, stars show achievement of a specific quality assessment. For clarification, please place the following amend beneath the table: “Quality assessment of non-randomized studies was done by using Newcastle Ottawa Quality Assessment for cohort studies. For Selection a maximum of four stars could be awarded. For a star in Representativeness the ERAS and non-ERAS groups had to consist of consecutive patients with urothelial cancer undergoing radical cystectomy. For Selection, patients had to be operated in the same institution. For Ascertainment of exposure perioperative outcomes had to be extracted from surgical and patient records. For Demonstration that outcome of interest was not present at start all studies were awarded with one point as perioperative outcome such as complication rate were the main outcomes.

For Comparability, two stars could be awarded. One star was given for type of radical cystectomy. If there was a significant difference in open, robotic or laparoscopic technique or in type of urinary diversion, no star was awarded. For the second star, groups had to be similar in BMI, comorbidities (measured in CCI, ASA-Score, or other similar score), T-Stage of urothelial cancer and age to be awarded with a star. For Outcome, Assessment of outcome had to be blind or assessed by patient, surgical or database records to be awarded with a star. For length of Follow-up, a length of 90days was considered adequate. A follow up above 90% was considered necessary due to the short time of necessary length of follow-up

**Table 5:** Summary of findings

Summary of findings
**ERAS compared to conventional care for radical cystectomy**
**Patient or population:** patients with bladder cancer **Setting:** hospital **Intervention:** radical cystectomy with ERAS protocol **Comparison:** radical cystectomy with conventional Care

Outcomes	Anticipated absolute effects^a*^ (95% CI)		Relative effect (95% CI)	№ of participants (studies)	Certainty of the evidence (GRADE)	Comments
	Risk with conventional Care	Risk with ERAS				
Length of stay (LOS)	The mean LOS ranged from 7—26 days	mean 3.46 days lower (4.94 lower to 1.98 lower)	–	3119 (2RCTs, 18 observational studies)	$\oplus\!\oplus\!\bigcirc\bigcirc$ LOW ^a,b^	The evidence suggests ERAS results in a reduction in LOS
Complication rate	597 per 1.000	529 per 1.000 (474–582)	OR 0.76 (0.61–0.94)	3373 (two RCTs, 19 observational studies)	$\oplus\!\bigcirc\!\bigcirc\!\,\bigcirc$ VERY LOW ^a^	ERAS may reduce/have little to no effect on complication rate, but the evidence is very uncertain
Time to defecation	The mean time to defecation ranged from 3.4–7.4 days	mean 1.38 days lower (2.06 lower to 0.69 lower)	–	1061 (two RCTs, six observational studies)	$\oplus\!\bigcirc\!\bigcirc\!\,\bigcirc$ VERY LOW ^a,b^	ERAS may reduce/have little to no effect on time to defecation, but the evidence is very uncertain
30d Readmission rate	210 per 1.000	163 per 1.000 (122–215)	OR 0.73 (0.52–1.03)	3215 (two RCTs, 15 observational studies)	$\oplus\!\bigcirc\!\bigcirc\!\,\bigcirc$ VERY LOW ^a^	ERAS seems to have no effect on readmission rate

*CI* confidence interval, *OR* odds ratio

**Explanations**

^a*^The risk in the intervention group (and its 95% confidence interval) is based on the assumed risk in the comparison group and the relative effect of the intervention (and its 95% CI)

^a^Inclusion of non-randomized studies

^b^High* I*^2^

**GRADE Working Group grades of evidence**

**High certainty:** we are very confident that the true effect lies close to that of the estimate of the effect

**Moderate certainty:** we are moderately confident in the effect estimate: The true effect is likely to be close to the estimate of the effect, but there is a possibility that it is substantially different

**Low certainty:** our confidence in the effect estimate is limited: The true effect may be substantially different from the estimate of the effect

**Very low certainty: **we have very little confidence in the effect estimate. The true effect is likely to be substantially different from the estimate of effect
